# Development of an Effective Nontoxigenic Clostridioides difficile–Based Oral Vaccine against C. difficile Infection

**DOI:** 10.1128/spectrum.00263-22

**Published:** 2022-05-18

**Authors:** Shaohui Wang, Duolong Zhu, Xingmin Sun

**Affiliations:** a Department of Molecular Medicine, Morsani College of Medicine, University of South Florida, Tampa, Florida, USA; Ohio State University

**Keywords:** nontoxigenic *Clostridioides difficile*, *Clostridioides difficile* infection, chimeric protein, vaccine, spore, oral immunization

## Abstract

The symptoms of Clostridioides difficile infection (CDI) are largely attributed to two *C. difficile* toxins, TcdA and TcdB. Significant efforts have been devoted to developing vaccines targeting both toxins through parenteral immunization routes. Recently, we generated a novel chimeric protein (designated Tcd169), comprised of the glucosyltransferase domain (GT), the cysteine protease domain (CPD), and the receptor binding domain (RBD) of TcdB, and the RBD of TcdA. Parenteral immunizations with Tcd169 provide mice effective protection against infection with a ribotype (RT) 027 *C. difficile* strain. In this study, we expressed Tcd169 in a nontoxigenic *C. difficile* CCUG37785 strain (designated NTCD), resulting in strain NTCD_Tcd169 to develop an oral vaccine that can target both *C. difficile* toxins and colonization/adhesion factors. Oral immunizations with NTCD_Tcd169 spores induced systematic and mucosal antibody responses against, not only both toxins, but also C. difficile flagellins (FliC/FliD). Intriguingly yet importantly, anti-Tcd169 sera raised against Tcd169 protein were significantly cross-reactive with FliC/FliD and two surface layer proteins (SlpA and Cwp2). Oral immunizations with NTCD_Tcd169 spores provided mice effective protection against infection with a hypervirulent RT027 *C. difficile* strain R20291and significantly reduced R20291spore numbers in feces compared with NTCD or PBS immunized mice. These results imply that the genetically modified, nontoxigenic *C. difficile* strain expressing Tcd169 may represent a novel mucosal vaccine candidate against CDI.

**IMPORTANCE** Clostridioides difficile is an enteric pathogen, and symptoms of C. difficile infection (CDI) are mainly by two exotoxins TcdA and TcdB. Active vaccination is cost-effective approach to prevent CDI and high rates of recurrence. Ideally, vaccines should target both C. difficile toxins and cell/spore colonization. In this study, we expressed immunodominant fragments of TcdA and TcdB (i.e., Tcd169) in a nontoxigenic *C. difficile* CCUG37785 strain, generating a promising oral/mucosal vaccine candidate against CDI, by targeting both toxins and colonization of pathogenic C. difficile strains. Importantly, anti-Tcd169 sera raised against Tcd169 protein were significantly cross-reactive with FliC/FliD and two surface layer proteins (SlpA and Cwp2), and all of which are involved in C. difficile adhesion/colonization *in vitro* and *in vivo*.

## INTRODUCTION

Clostridioides difficile (C. difficile) is an anaerobic, spore-forming, and toxin-producing Gram-positive bacterium and was identified as the leading cause of antibiotic-associated diarrhea and colitis in 1978 (1). Symptoms of C. difficile infection (CDI) are mainly caused by two large protein toxins, toxin A (TcdA) and toxin B (TcdB) (2, 3). TcdA and TcdB share similar domain structures, including the N terminus catalytic glucosyltransferase domain (GT), the autoproteolytic cysteine proteinase domain (CPD), the central translocation domain (TM), and the C-terminal receptor-binding domain (RBD) (4). Currently, CDI has become one of the most significant nosocomial infections (5), occurring worldwide (6). Very few antibiotics are available for the treatment of CDI (7), and none of them is fully effective with increased risk of prolonged diarrhea and high rates of recurrent CDI (rCDI) up to 36% (5, 8). Vaccination is considered a cost-effective and promising approach for the treatment or prevention of CDI and rCDI, as it would not disrupt the bacterial balance of the host (9).

Currently, there are two C. difficile vaccine candidates at different stages of clinical trials, including a fusion protein vaccine (VLA84) from Valneva, and a genetically modified TcdA and TcdB from Pfizer (10). VLA84 contains RBD domains of TcdA and TcdB and lacks the immunodominant GTD domain of TcdB. The Pfizer vaccine still requires chemical inactivation to abolish residual toxic activity. These vaccine candidates use intramuscular (IM) injections for immunization and only target toxins. However, vaccines should target both C. difficile toxins and colonization to prevent disease and reduce recurrence and transmission. In addition, C. difficile is an enteric pathogen, and mucosal/oral immunization would be particularly useful to protect the host against CDI considering that the gut is the main site of disease onset and progression. Considering the recent failure of the Sanofi Pasteur vaccine candidate (11), which is based on inactivated TcdA and TcdB administered IM a parenteral immunization might not be suitable for a mucosal pathogen. Therefore, it should be preferable to generate an oral/mucosal vaccine to induce local mucosal and systemic anti-toxin and anti-C. difficile colonization responses. In fact, mucosal anti-toxin IgA Ab from CDI patients has toxin-neutralizing activity (12), and mucosal anti-toxin Ab is required in hamsters for efficient protection after toxoid immunizations (13). Indeed, we and others have shown that orally delivered live bacterial vaccines expressing C. difficile toxin fragments can elicit both intestinal IgA and systemic IgG antibodies, both of which can neutralize toxins, and protect animals from lethal C. difficile toxin or spore challenge (14–16).

Oral vaccination provides both social and economic advantages, especially in developing countries, for the use of needle-free vaccine administration (17, 18). Previously, we expressed mTcd138 in the nontoxigenic strain C. difficile CCUG37785 (designated NTCD) as a vaccine against CDI (14, 19). Fusion protein mTcd38 that is comprised of the GT and CPD of TcdB and the RBD of TcdA. Recently we reported that NTCD has higher adhesion and sporulation capability compared with R20291 strain, and that oral inoculation of NTCD spores prior to infection with R20291 spores provided mice nearly full protection against CDI (19). Recently, we generated an enhanced fusion protein antigen Tcd169 that is comprised of GT, CPD, and RBD of TcdB and RBD of TcdA. Tcd169 immunization induced protective immunity against TcdA/TcdB challenge in mice and also provided mice full protection against infection with a hyper-virulent *C. diﬃcile* strain (14). In this study, we expressed Tcd169 in NTCD, generating the strain NTCD_Tcd169 as an oral vaccine.

## RESULTS

### Expression of Tcd169 in the nontoxigenic C. difficile CCUG37785 strain.

**FIG 1:**
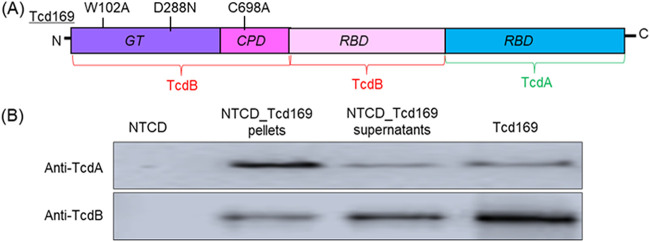
Expression of Tcd169 in the nontoxigenic *C. difficile* CCUG37785 (designated NTCD). (A) Tcd169 was constructed by fusing the glucosyltransferase domain (GT), the cysteine proteinase domains (CPD) and the receptor binding domain (RBD) of TcdB with the RBD of TcdA. Two-point mutations were made in the GT of TcdB and one point mutation was made in the CPD of TcdB, which essentially eliminates the toxicity of Tcd169. (B) Western blot analysis of Tcd169 expression in supernatants and pellets of strains NTCD_Tcd169 and NTCD using anti-TcdA or anti-TcdB antibodies.

Previously, we fused GT, CPD, and RBD of TcdB and RBD of TcdA, resulting in Tcd169 (14) (Fig. 1A). To ensure that Tcd169 is atoxic, point mutations were made in GT (W102A, D288N) and CPD (C698A) of TcdB. C. difficile CCUG37785 (designated NTCD) is a nontoxigenic strain characterized by us recently (14, 19). The gene sequence coding for Tcd169 was cloned into the E. coli–C. difficile shuttle vector pRPF144, and transconjugated into NTCD, generating strain NTCD_Tcd169. Western blot analysis showed that Tcd169 was expressed in NTCD_Tcd169, and was detected both intracellularly and in the supernatant of the bacterial culture (Fig. 1B).

### Oral immunization with NTCD_Tcd169 spores induces mucosal and systemic antitoxin antibody responses in mice.

**FIG 2:**
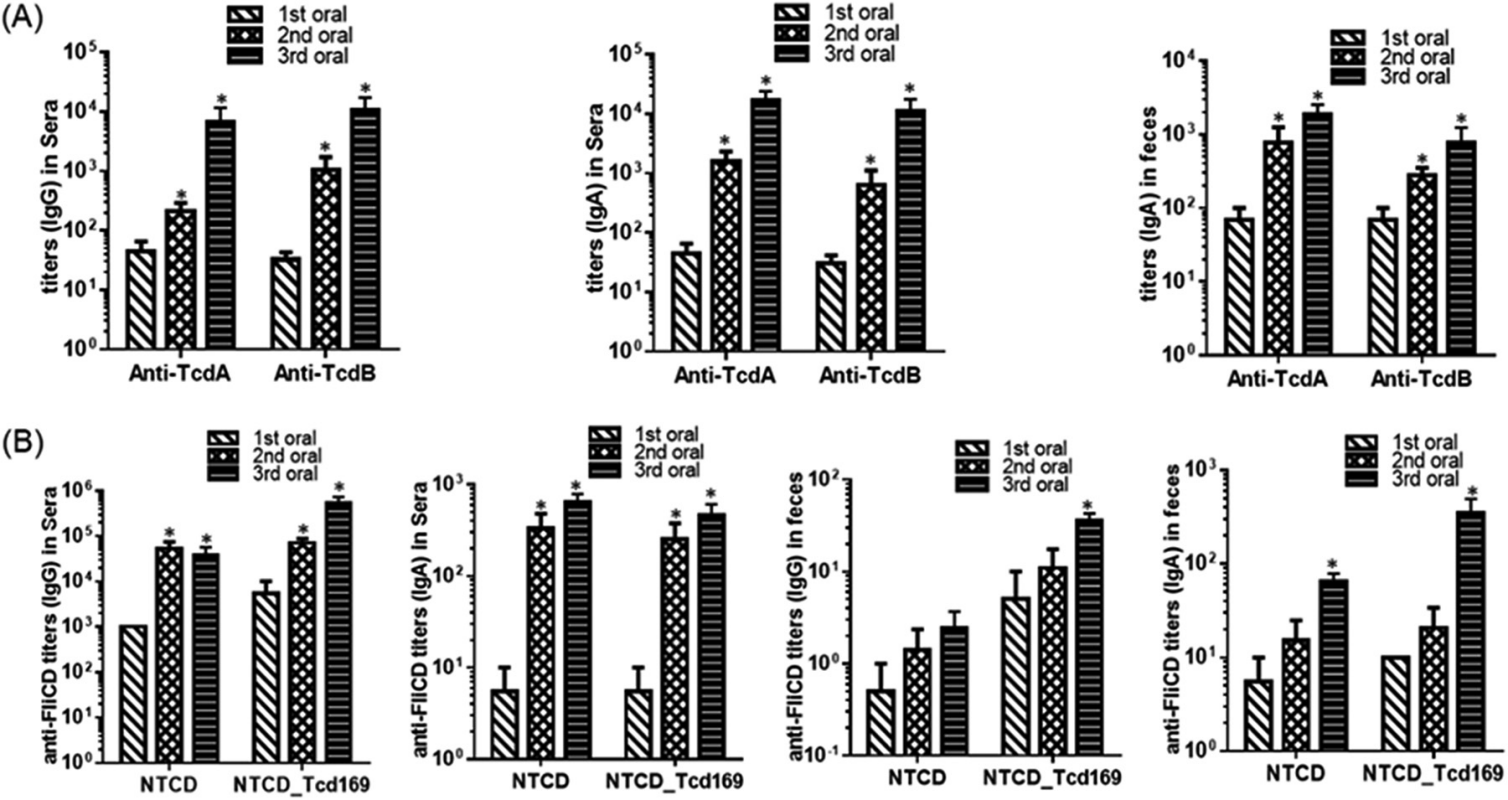
Oral immunization (IM) of mice with NTCD_ Tcd169 spores induced protective immune responses. (A) Oral immunization of mice with NTCD_Tcd169 spores induced mucosal and systemic toxin-specific antibody responses. Groups of C57 BL/6 mice (*n* = 10) were orally immunized with NTCD_Tcd169 (2 × 10^6^ spores/immunization, 3 times at 12-day intervals). Sera and feces were collected after each immunization. Before use, feces were dissolved (0.1g/mL) in PBS containing proteinase inhibitors. Anti-TcdA/anti-TcdB IgG titers in sera and anti-TcdA/anti-TcdB IgA titers in sera or in feces were determined by ELISA (*, *P* < 0.05 versus 1st oral). (B) Oral immunization of mice with NTCD_Tcd169 or NTCD spores induced mucosal and systemic antibody responses against FliCD. Sera and feces were collected after each immunization. Anti-FliCD IgG or IgA titers in sera or feces were determined by ELISA (*, *P* < 0.05 versus 1st oral).

Immunization with NTCD_Tcd169 (2 × 10^6^ spores per immunization for 3 times at 12-day intervals, by gavage) induced significant toxin-specific antibody responses in both sera and fecal samples (Fig. 2A).

**FIG 3:**
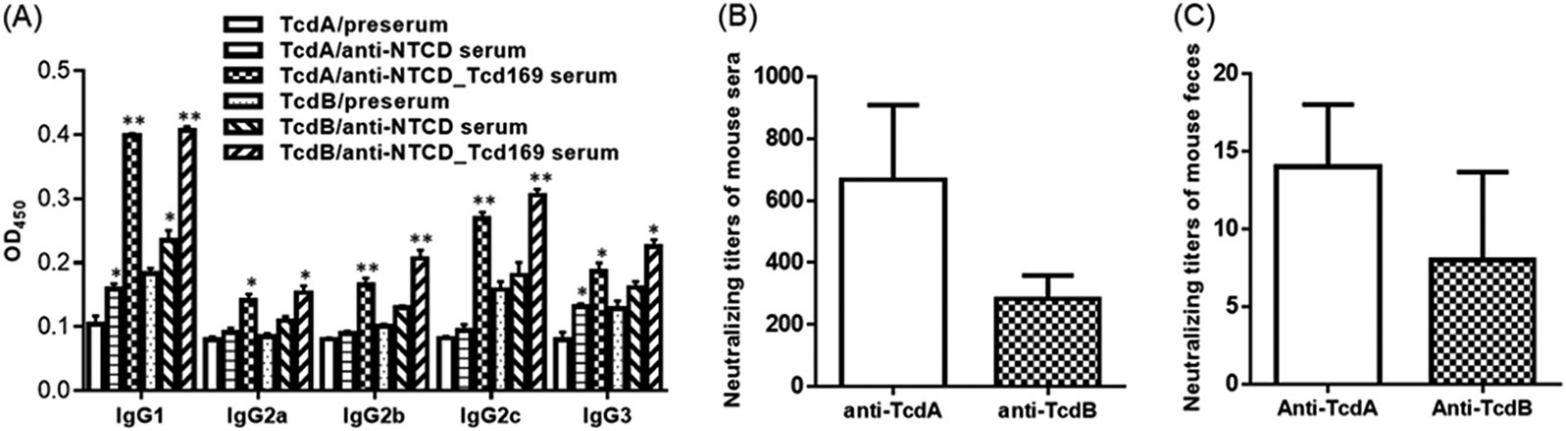
Antitoxin IgG isotypes and antitoxin neutralizing titers of sera from mice orally immunized with NTCD_Tcd169 spores. Mice were immunized with NTCD or NTCD_Tcd169 spores at 2 × 10^6^ spores/mouse three times, and serum samples were collected. (A) Antitoxin IgG isotypes of the sera were measured by standard ELISAs. OD450, optical density at 450 nm. Antitoxin-neutralizing titers of sera (B) and feces (C) from mice orally immunized with NICD_Tcd169 mice. (*, *P* < 0.05 and **, *P* < 0.01 compared with presera).

To determine the nature of immune responses (i.e., Th1 or Th2) elicited by NTCD_Tcd169 immunization, we measured isotypes of anti-TcdA/anti-TcdB IgGs. At a dilution of 1 × 10^3^, anti-NTCD_Tcd169 sera showed significantly higher levels of IgG1, IgG2b, IgG2c, IgG2a, and IgG3 subclass anti-toxin antibodies, with IgG1 being the most dominant subclass compared with presera, indicating that NTCD_Tcd169 immunizations can induce both Th1 and Th2 responses (Fig. 3A). The *in vitro* toxin-neutralizing activity of anti-NTCD_Tcd169 sera was also tested, revealing that NTCD_Tcd169 immunization induced neutralizing antibodies against both toxins, with anti-TcdA titers being significantly higher than anti-TcdB titers in sera (Fig. 3B). Significant toxin-neutralizing activities were also detected in feces of NTCD_Tcd169 immunized mice (Fig. 3C).

### Oral immunization of mice with NTCD_Tcd169 spores induces mucosal and systemic immune responses against C. difficile surface proteins.

**FIG 4:**
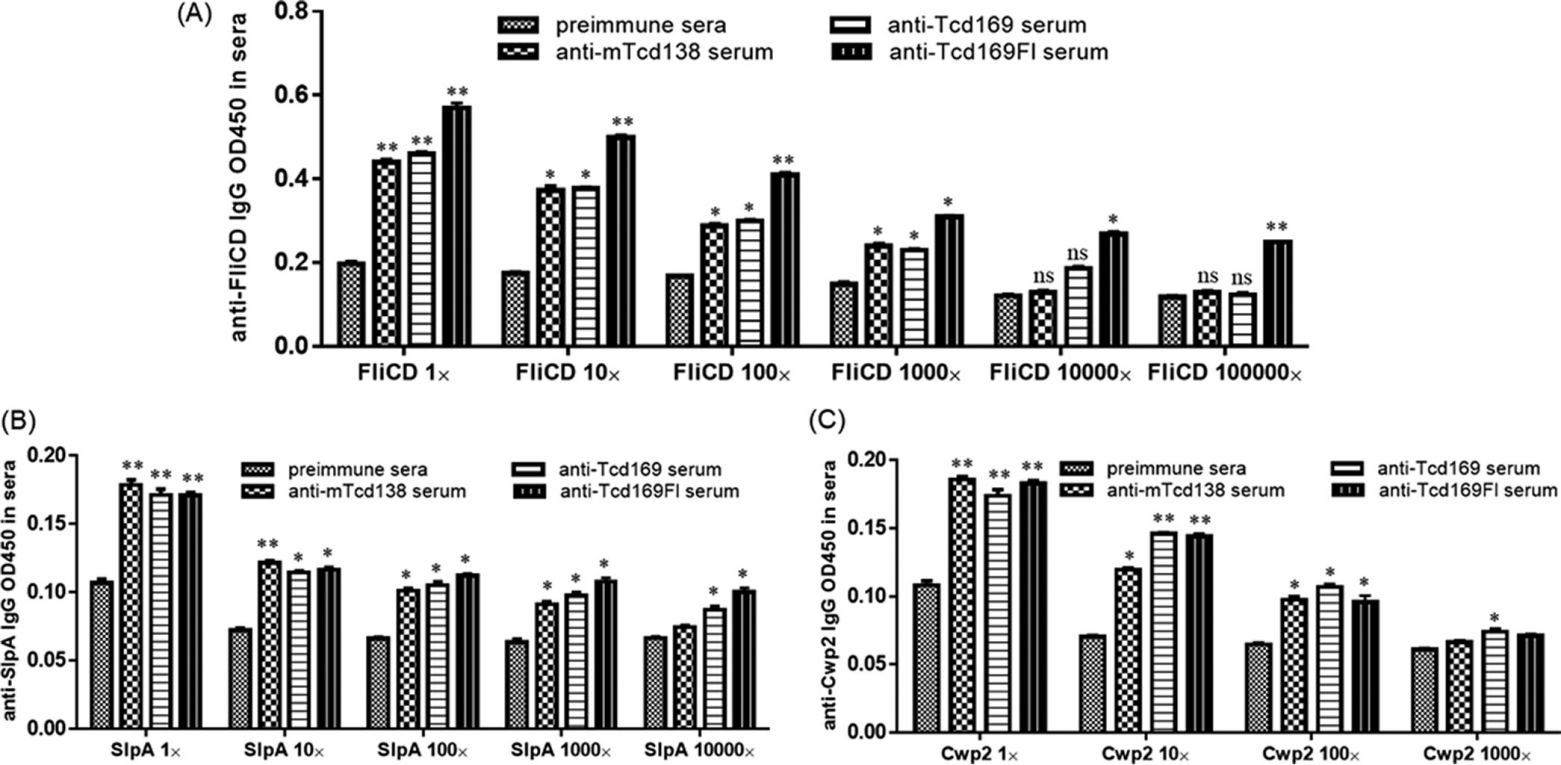
Anti-Tcd169, anti-mTcd138 and anti-Tcd169FI sera cross-reacts with C. difficile surface proteins. Immune sera were collected after 3rd immunizations (i.p.) of mice with 10 μg of Tcd169, mTcd138 or Tcd169FI in the presence of alum in this work or previously Across reactivities of anti-Tcd169, anti-mTcd138 and anti-Tcd211 sera with C. difficile surface proteins FliCD (A), SlpA (B), and Cwp2 (C) were determined by ELISAs (*, *P* < 0.05 and **, *P* < 0.01 versus preimmune sera).

To determine whether NTCD or NTCD_Tcd169 immunization can induce anti-C. difficile responses, we measured anti-FliCD antibody levels in mouse sera and feces. FliCD is a fusion protein containing the full length of FliC and FliD C. difficile flagellins (14). Interestingly, we found that NTCD_Tcd169 immunization could induce higher levels of anti-FliCD IgG (*P* = 0.0016 for the 3rd immunization) and IgA (*P* = 0.07 for the 3rd immunization) antibodies in feces, and higher levels of anti-FliCD IgG antibodies (*P* = 0.06 for the 3rd immunization) in sera in comparison with NTCD immunization (Fig. 2B). This observation agrees with our previous finding that NTCD_mTcd138 immunization induced higher levels of anti-FliCD IgG/IgA responses in both sera and feces compared with NTCD immunization (14). Protein mTcd138 is comprised of the GTD and CPD domains of TcdB and the RBD of TcdA with two-point mutations in the GTD of TcdB to knock out toxicity. We reasoned that anti-Tcd169 and anti-mTcd138 antibodies might cross-react with FliCD possibly due to the potential conformational epitopes since there are no sequence similarities between Tcd169/mTcd138 and FliCD. To this end, we determined the cross-reactivities of anti-Tcd169, anti-mTcd138 and anti-Tcd169FI sera with FliCD by ELISAs. Sera were collected from mice after the 3rd immunizations in this work or previously (20, 21). Tcd169FI was generated previously by fusing Tcd169 with *S. typhimurium* flagellin (sFliC) (20). Indeed, anti-Tcd169, anti-mTcd138 and anti-Tcd169FI sera displayed significant cross-reactivity with FliCD at a 1000-fold dilution, and anti-Tcd169FI sera also displayed significant cross-reactivity with FliCD at a 10^5^ dilution, which could be caused by the similarity between FliC proteins from both *S. typhirium* and C. difficile species or by sFliC-mediated immunogenicity enhancement of antigen (Fig. 4A). More interestingly, anti-Tcd169, anti-mTcd138 and anti-Tcd169FI sera were also significantly cross-reactive with SlpA and Cwp2 (Fig. 4B), two abundant C. difficile surface layer proteins (22–25).

### Immunization with NTCD_Tcd169 spores provides mice effective protection against challenge with an epidemic and hyper-virulent C. difficile strain.

**FIG 5:**
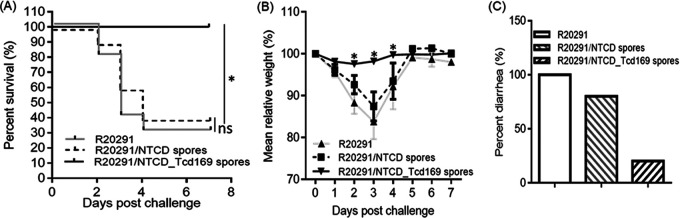
Oral immunization of mice with NTCD_Tcd169 spores provides mice full protection against infection with *C. difficile* strain R20291. Mice were challenged with *C. difficile* R20291 spores (10^6^/mouse) 14 days after the third oral immunization with NTCD spores, NTCD_mTcd169 spores or PBS. Kaplan-Meier survival plots (A), mean relative weight of all surviving mice (up to the day of death) (B) of different groups, and frequency of diarrhea (C) are illustrated. Data were presented as mean relative weight ± standard error.

We further evaluated protection efficacy of NTCD_Tcd169 in a mouse model of CDI. After three immunizations by gavage, mice were orally infected with 10^6^ C. difficile R20291 spores. In PBS-immunized group (control), mice developed significant disease symptoms as demonstrated by significant weight loss (Fig. 5B) and severe diarrhea (Fig. 5C) with 70% mortality by day 4 postinfection (Fig. 5A). Conversely, NTCD_Tcd169-immunized group was fully protected against CDI-induced death and weight losses (Fig. 5A and B). Only 10% of the mice showed slight diarrhea (Fig. 5C). Interestingly, NTCD immunization showed slight, but not significant protection against C. difficile R20291 challenge (Fig. 5).

### Immunization of mice with NTCD_Tcd169 spores, decrease the amount of spores and toxins in the feces.

**FIG 6:**
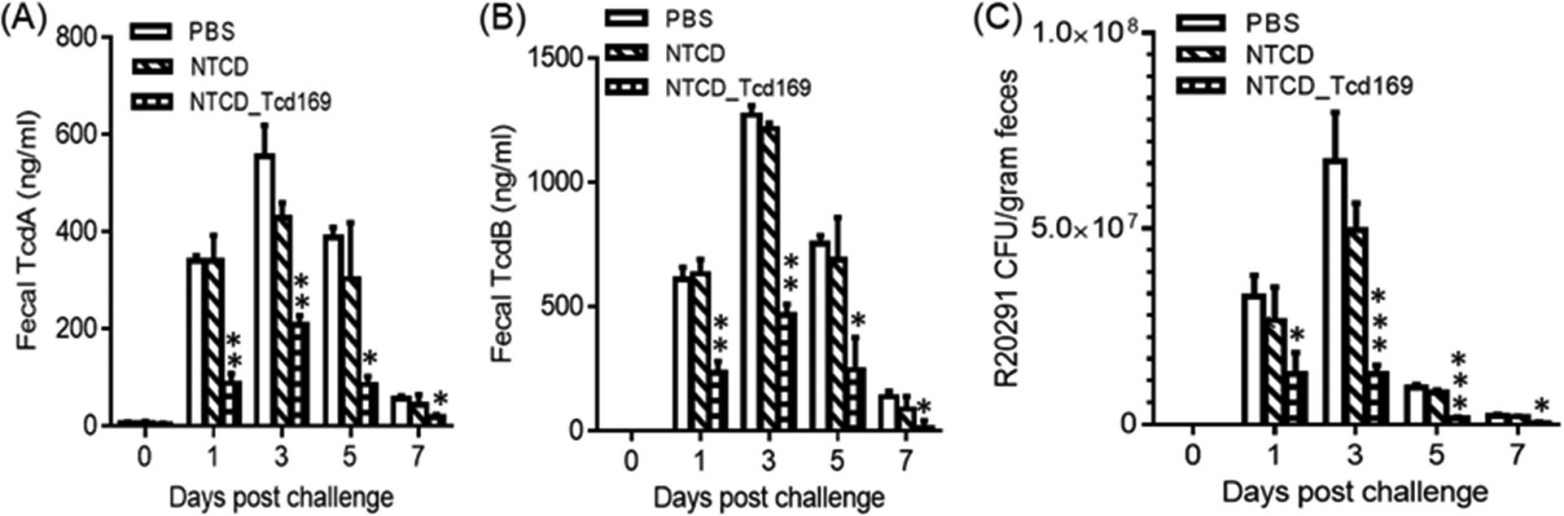
Oral Immunizations of mice with NTCD_Tcd169 decrease the C. difficile spores and toxins in the feces after CDI. TcdA (A) or TcdB (B) levels in feces were determined by ELISA. (C) Fecal samples were collected for R20291 spore enumeration. The *tcdB* gene was amplified to distinguish toxigenic C. difficile R20291 and nontoxigenic C. difficile strain (NTCD). Bars stand for means ± SD. (*, *P* < 0.05, **, *P* < 0.01, ***, *P* < 0.001, NTCD_Tcd169 versus PBS or NTCD). In addition, in (C) day 1 (ns, PBS versus NTCD; ns, NTCD versus NTCD_Tcd169), day 3 (ns, PBS versus NTCD; **, *P* < 0.01, NTCD versus NTCD_169), day 5 (ns, PBS versus NTCD; **, *P* < 0.01, NTCD versus NTCD_169), day 7 (ns, PBS versus NTCD; **, *P* < 0.01, NTCD versus NTCD169).

NTCD_Tcd169-immunized mice secreted significantly smaller amounts of TcdA (Fig. 6A) and TcdB (Fig. 6B) in the feces, compared to NTCD or PBS immunization groups (Fig. 6A and B). The fecal samples of NTCD-immunized mice contained less, but not statistically significant R20291 spores compared to the PBS group. However, fecal samples of NTCD_Tcd169-immunized mice contained significantly less R20291 spores compared to NTCD-only or PBS immunization groups (Fig. 6C). Furthermore, NTCD_Tcd169 immunized mice secreted significantly (*P* < 0.01) less R20291 spores in comparison with NTCD immunized mice on days of 3, 5, and 7 postinfection.

## DISCUSSION

C. difficile is intrinsically resistant to many antibiotics, limiting treatment options (26). This problem is compounded by the dissemination of hypervirulent strains such as R20291 (BI/NAPI/027) (27–29). The current treatment options of C. difficile infection (CDI) including metronidazole, vancomycin and fidaxomicin are not fully effective (30, 31) with a recurrence rate of 15–35% (32, 33). Alarmingly, emergence of resistant C. difficile strains to these antibitoics has been frequently reported (34). Consequently, C. difficile is classified as an “urgent antibiotic resistant threat” by the Centers for Disease Control and Prevention (35, 36). Symptoms of CDI are mainly caused by two toxins TcdA and TcdB. Active vaccination is generally accepted as a logical and cost-effective approach to prevent CDI (37), but no vaccine effective at preventing CDI is licensed (10, 38, 39).

Per our (14) and others’ previous studies (40–42), nontoxigenic C. difficile is safe and had no major adverse effects when given to healthy subjects with or without antibiotics (40). Previously, we have engineered NTCD to express mTcd138, generating a promising oral vaccine candidate against CDI (14). Recently, we also generated a new chimeric protein (Tcd169) vaccine candidate by fusing GT, CPD, and RBD of TcdB and RBD of TcdA (14). To develop mucosal vaccines that can induce immune responses not only against toxins and but also *C. difficile* colonization, we engineered NTCD to express Tcd169, generating the strain NTCD_Tcd169. The data presented here demonstrates that oral immunization of mice with NTCD_Tcd169 spores induced not only IgG/IgA antibody responses specific for both toxins in sera, as well as IgA antibodies specific for both toxins in feces (Fig. 2A), but also antibody responses to *C. difficile* flagellins (FliC and FliD) (Fig. 2B), suggesting that NTCD_Tcd169 immunization could induce a protective antibody response to colonization factors of *C. difficile*.

Surprisingly, we found that NTCD_Tcd169 could induce stronger immune responses to FliCD than NTCD only did. For the first time, it was established that anti-Tcd169, anti-mTcd138 and Tcd169FI sera were significantly cross-reactive not only to FliCD but also toward C. difficile surface proteins (SlpA and Cwp2). This finding is both scientifically and clinically significant toward developing C. difficile vaccines and immunotherapies. Cwp2 is a cell wall protein (CWP) associated with the surface layer proteins of C. difficile. Cwp2 contains the cell wall binding 2 (CWB2) domain, characteristic of cell wall binding proteins, which is responsible for attachment to the bacterial cell. As such it is possible that Cwp2 is an attachment protein with a role in colonization of host tissues. This protein has been shown to be conserved and abundant on C. difficile cell surface. It seems that all patients with CDI have antibodies against Cwp2, suggesting that it may be an effective vaccine target (22). The gene *slpA* has a conserved genomic location among all C. difficile strains, and encoded a precursor protein SlpA, which has three subdomains: an N-terminal secretion signal, a highly variable low molecular weight (LMW) region, and a highly conserved high molecular weight (HMW) region containing three tandem CWB2 motifs (25, 43). After synthesis, the precursor protein is cleaved by proteinase Cwp84 to generate HMW-SLP and LMW-SLP (44). Both HMW- and LMW-SLPs are linked by noncovalent interactions (23, 45). HMW-SLPs share up to 97% sequence identity between C. difficile strains (46), and can specifically bind to gastrointestinal tissues and human epithelial cells (47). Our data showed that fecal samples from NTCD/NTCD_Tcd169-immunized mice contained less R20291 spores than the control. Furthermore, NTCD_Tcd169 immunized mice secreted significantly (*P* < 0.01) less R20291 spores in comparison with NTCD immunized mice. Taken together, our data suggest that Tcd169 antigen induces protective immune responses against both C. difficile toxins and colonization factors.

In agreement with our finding, another recent study showed that antibodies against a fragment of TcdA (TcdA_26-39_) RBD (but not to toxoids), whether raised to the recombinant protein or to TcdA_26-39_ expressed on the B. subtilis spore surface, cross-react with several seemingly unrelated proteins expressed on the vegetative cell surface or spore coat of C. difficile, including two dehydrogenases, AdhE1 and LdhA, as well as the CdeC spore protein. Another nontoxigenic strain engineered to express a fragment of TcdB induced a humoral immune response in mice not only against TcdB but also against a surface lipoprotein of C. difficile (personal communication with Ruth Griffin). This cross-reaction is intriguing yet important and indicates the important roles of conformational immuno-epitopes in inducing broad and strong immune responses.

IgG1 antibody production is associated with Th2 response, and IgG2a, IgG2b, IgG2c, and IgG3 antibodies are associated with Th1 response (48, 49). NTCD_Tcd169 immunizations can induce both Th1 and Th2 responses (Fig. 3A). Anti-toxin antibodies may enhance toxin toxicity (50). Our data showed that immunizations with NTCD_Tcd169 spores can induce potent neutralizing antibodies against both TcdA and TcdB (Fig. 3B and C). Taken together, NTCD_Tcd169 is a promising oral vaccine candidate against CDI.

## MATERIALS AND METHODS

### Animals.

All studies followed the Guide for the Care and Use of Laboratory Animals of the National Institutes of Health and were approved by the Institutes Animal Care and Use Committee (IACUC) at the University of South Florida. Wild-type C57BL/6 mice were purchased from Charles River Laboratories.

### Expression of fusion protein Tcd169 in the nontoxigenic strain CCUG37785 (designated NTCD).

Previously, we constructed a recombinant fusion protein Tcd169, which contains the GT, CPD, and RBD of TcdB and RBD of TcdA. The synthesized Tcd169-encoding sequence was amplified using pMA-Tcd169 as the template (synthesized by Genscript) and primers F169 (CCCGAGCTCCTGCAGTAAAGGAGGAAATTTTATGAGTTTAGTTAATA GAAAACAG) and R169 (CGCGGATCCTTACCCATATATCCCAGGGGCTTTTAC). The fragment was cloned into pRPF144 (43) (kindly provided by Robert Fagan from University of Sheffield) using SacI and BamHI sites. Then the chimeric DNA encoding Tcd169 was introduced into the NTCD by conjugation as previously described (51), generating strain NTCD_Tcd169. Strain NTCD was originally from Michel Delmée (UCLouvain, Belgium) who deposited the strain to the culture collection of the University of Gothenburg (CCUG), Sweden (14).

### Expression and purification of C. difficile proteins SlpA, Cwp2 and FliCD.

Gene sequences encoding FliC and FliD from C. difficile R20291 were bridged with a linker (ggt ggc tct ggt) sequence, synthesized by Geneart (Germany) and cloned into BsrGI and EagI restriction sites of pHis1525 (52). FliCD was expressed in B. megaterium and purified as described previously (14). The *slpA* gene encoding the high-molecular-weight (HMW) SlpA from C. difficile R20291 was cloned into NdeI and XhoI restriction sites of pET28a using forward primer (tacgcatatggctgcaaaggcttcaatt) and reverse primer (tacgctcgagttacatacttaataaatctttta). The HMW SplA protein with a N-terminal His tag was purified by Ni-affinity chromatography. The predicted nontransmembrane (aa 29–317) encoding sequence of *cwp2* gene from C. difficile R20291 strain was cloned into NheI and XhoI restriction sites of pET28a using forward primer (agatgctagccaggtaaaaaaagaaacaataac) and reverse primer (ggtgctcgagttattctaatgcagctttggcat). The Cwp2 protein fragment with a N-terminal His tag was purified by Ni-affinity chromatography.

### Western blotting.

NTCD strains were grown in BHI medium in an anaerobic chamber at 37°C for 24–48 h, after which culture supernatants and vegetative cell pellets were collected. C. difficile vegetative cell pellets were lysed in protein lysis buffer (dH_2_O, 0.05 M Tris, 0.3 M NaCl, 0.5% TTX 100, 2 mM EDTA, 0.4 mM Na_3_VO_4_, 2.5 mM Leupeptin, 2.5 mM Aprotinin, 2.5 mM 4-Nitrophenyl 4-guanidinobenzoate hydrochloride [NPGB]). Protein concentration was measured using a BCA protein assay (Thermo Scientific, Suwanee, GA). Protein extracts were subjected to 12% SDS-PAGE separation. Then, proteins were transferred onto a Nylon membrane. After blocking for 1 h at room temperature with 5% skim milk, the membrane was incubated overnight at 4°C with anti-toxin A and anti-toxin B antibodies (1:1000). After washing with PBST (PBS with 0.05% Tween 20), the membrane was incubated with horseradish peroxidase-conjugated secondary goat anti-mouse antibody (Cat: ab97023, IgG, 1:3000, Abcam, Cambridge, MA), the antibody-reactive bands were revealed by enhanced chemiluminescence detection on Hyperfilm (Thermo Fisher Scientific, Waltham, MA).

### Preparation of *C. difficile* spores.

Sporulation of the *C. difficile* R20291, NTCD, and NTCD_Tcd169 strains was induced in Clospore medium as described previously (14, 53). Briefly, an overnight 20 mL *C. difficile* cultured in Columbia Broth was inoculated into 500 mL of Clospore medium and incubated for 1–2 weeks at 37°C in an anaerobic incubator. The spore suspension was centrifuged at 10000g for 20 min, and the pellet was washed 5 times with sterile water and suspended in 10 mL of ddH_2_O. The spore suspension was layered onto the top of 10 mL of 50% (wt/vol) sucrose in water in a 15-mL tube. The gradient was centrifuged at 3200 × *g* for 20 min, after which the spore pellet at the bottom was washed five times to remove the sucrose and was resuspended in water. All spore preparations were >99% pure, free of vegetative cells and debris (54). The spore concentration was determined by serial dilution on TCCFA or BHI plates.

### Mouse immunization and mouse model of C. difficile infection.

Female C57/BL6 mice were housed under the same conditions at a seminatural light cycle of 14 h:10 h (light: dark) in a specific pathogen-free (SPF) environment. During immunizations and infection with C. difficile, mice were housed in infection rooms. Mice (*n* = 10) were immunized 3 times at 12-day intervals via oral administration with 2 × 10^6^/100 μL spores of NTCD or NTCD_mTcd169. Control mice received the same volume of PBS. Sera were collected, and anti-TcdA/TcdB IgG titers were determined by ELISA. Seven days after the third immunization, immunized or control mice were given drinking water containing a mixture of six antibiotics, including ampicillin (200 mg/kg), kanamycin (40 mg/kg), gentamicin (3.5 mg/kg), colistin (4.2 mg/kg), metronidazole (21.5 mg/kg) and vancomycin (4.5 mg/kg) for 4 days, and then received autoclaved water for 2 days, followed by a single dose of clindamycin (10 mg/kg) intraperitoneal injection before challenge with 10^6^ C. difficile R20291 spores/mouse via oral gavage as described previously (55). After infection, mice were monitored daily for a week for survival, weight changes, diarrhea, and other symptoms of the disease. Diarrhea was defined as wet tails, loosen or watery feces. Death included the numbers of mice died after infection and mice euthanized when weight loss was > 20%.

### ELISA for determining anti-Toxin/FliCD IgA and IgG titers.

ELISAs were performed as previously described (56). Briefly, Costar 96-well ELISA plates were coated with 100 μL/well of TcdA (0.5 μg/mL), TcdB (0.5 μg/mL), or FliCD (0.5 μg/mL) at 4°C overnight. Following one wash of the unbound material with PBS, plates were blocked with 300 μL of blocking buffer (PBS + 5% dry milk) at room temperature for 2 h. After one wash with PBS, 100 μL of 10-fold diluted sera or fecal samples were added into each well of the plates and incubated for 1.5 h at room temperature. Following one wash with PBS, 100 μL of mouse IgG-HRP (1:3000) or mouse IgA-HRP (1:3000) were added to each well and incubated for 30 min to 1 h. After another wash with PBS, substrate TMB was added to allow color development at room temperature for 5–30 min. The reaction was stopped by addition of H_2_SO_4_ to each well, and the OD values at 450 nm were recorded by a spectrophotometer. Anti-toxin and anti-FliCD IgG or IgA titers of a given sample (serum or fecal sample from immunized mice was defined as the dilution factor at which the OD_450nm_ is greater or equal to 2-fold that of serum or fecal sample from nonimmunized mice).

### ELISA for determining across reactivities of anti-Tcd169, anti-mTcd138 and anti-Tcd169FI sera with C. difficile proteins FliCD, SlpA and Cwp2.

ELISAs were performed as described above. Briefly, Costar 96-well ELISA plates were coated with 100 μL/well of FliCD (0.5 μg/mL), SlpA (0.5 μg/mL) or Cwp2 (0.5 μg/mL) at 4°C overnight. Following washing of the unbound material, plates were blocked with 300 μL of blocking buffer (PBS + 5% dry milk) at room temperature for 2 h. After washing, 100 μL of 10-fold diluted presera, anti-mTcd138 sera, anti-Tcd169 sera and anti-Tcd169FI serum samples were added into each well of the plates and incubated for 1.5 h at room temperature. Following washing with PBS, 100 μL of mouse IgG-HRP or IgA-HRP (1:3000) were added to each well and incubated for 30 min to 1 h. After a washing step with PBS, substrate TMB was added to allow color development at room temperature for 5–30 min. The reaction was stopped by addition of H_2_SO_4_ to each well, and the OD values at 450 nm were recorded by a spectrophotometer.

### Neutralizing assays.

Mouse intestinal epithelial CT26 cells were used to assess the *in vitro* neutralizing activity of serum samples. The neutralizing titer is defined as the maximum dilution of the samples that blocks cell rounding caused by toxin at a given concentration. This given concentration is the minimum dose of the toxin that causes all cells to round after a 24-h exposure to the toxin, i.e., 2.5 and 0.1 ng/mL for TcdA and TcdB, respectively.

### Measurement of antitoxin IgG Isotypes.

IgG1, IgG2a, IgG2b, IgG2c, and IgG3 anti-TcdA/B concentrations in the sera of Tcd169 spores-immunized mice were determined by ELISA using biotinylated anti-mouse IgG isotype antibodies.

### Quantification of C. difficile spores from mouse feces.

Fecal samples were collected on days 0, 1, 3, 5, and 7 postinfection. 50 mg of feces were dissolved with 500 μL sterile water for 16 h at 4°C, and then treated with 500 μL of 95% ethanol (Sigma-Aldrich) for 1 h at room temperature to kill vegetative cells. Samples were vortexed, serially diluted and plated onto selective medium supplemented with taurocholate (0.1% wt/vol), Cefoxitin (8 μg/mL), d-cycloserine (250 μg/mL). The plates were incubated anaerobically at 37°C for 48 h, counted and the results were expressed as the CFU/gram of feces. The TcdB gene was amplified by colony PCR to distinguish toxigenic *C. difficile* R20291 and nontoxigenic *C. difficile* strains.

### Quantitation of C. difficile toxins in mouse feces.

After challenge with *C. difficile* spores, feces were collected, and dissolved in PBS (0.1g/mL) containing a protease inhibitor cocktail, and the supernatants were collected after centrifugation, and used for determination of TcdA/TcdB concentrations by ELISA. Briefly, 96-well Costar microplates were coated with 100 μL of anti-TcdA antibody (1 μg/mL) and anti-TcdB antibody (1 μg/mL) overnight in PBS at 4°C. The next day, each well was blocked with 300 μL of blocking buffer (PBS + 5% dry milk) at RT for 2 h. Next, standards and samples were added to each well (100 μL) in duplicate, and incubated for 90 min at 25°C. Following a wash with PBS, HRP-chicken anti–C. difficile TcdA/TcdB (1:5,000 dilution in PBS, Gallus Immunotech, Shirley, MA) was added to wells for 30 min at RT. After three washes with PBS, the TMB microwell peroxidase substrate was added for 20 min at RT in the dark. The reaction was stopped with 2 N H_2_SO_4_, and the absorbance was measured using a plate reader at 450 nm.

### Statistical analysis.

Animal survival curves were analyzed by Kaplan-Meier survival analysis with a log-rank test of significance. Data between two groups were analyzed by unpaired Student's *t* test. Data between more than two groups were analyzed by one-way analysis of variance (ANOVA) with *post hoc* analysis by Bonferroni tests. Data are expressed as means ± standard errors of means. Differences were considered statistically significant when *P* < 0.05 (*). All statistical analyses were performed using GraphPad Prism software.
